# Effects of Aerobic Training in Patients with Subacute COVID-19: A Randomized Controlled Feasibility Trial

**DOI:** 10.3390/ijerph192416383

**Published:** 2022-12-07

**Authors:** Stefano Corna, Marica Giardini, Marco Godi, Lucia Bellotti, Ilaria Arcolin

**Affiliations:** Istituti Clinici Scientifici Maugeri IRCCS, Division of Physical Medicine and Rehabilitation of Veruno Institute, 28013 Gattico-Veruno, Italy

**Keywords:** aerobic exercise, COVID-19, feasibility, physical therapy, subacute COVID-19, randomized controlled trial, rehabilitation, sequelae

## Abstract

Many clinical practice recommendations indicate rehabilitation as essential for patients with sequelae of severe or critical COVID-19 and suggest the prompt initiation of a multicomponent rehabilitation program focused on aerobic and endurance training. However, randomized controlled trials (RCTs) regarding aerobic exercise are lacking. Therefore, we aimed to assess the feasibility and effectiveness of the addition of aerobic training to standard rehabilitation in subjects with subacute COVID-19. Participants were 32/214 patients with the sequelae of severe or critical COVID-19 in the acute phase who were eligible and agreed to participate in the study (eligibility = 15%, recruitment = 100%). After randomization and assessment with functional and strength tests, all the participants underwent an inpatient-tailored rehabilitation program (50 min/day, 5 days/week, 10 sessions); in addition, the experimental group performed a low- to moderate-intensity aerobic exercise (30 min/day, 10 sessions). No dropouts or severe adverse events were reported, with an attendance rate of 95.6%. Most of the secondary outcomes significantly improved in both groups, but the improvement in the Functional Independence Measure and Cumulated Ambulation Score—Italian version was significantly greater in the experimental group (at least, *p* < 0.05). This RCT showed that aerobic exercise is feasible and safe in subacute COVID-19. Moreover, it appears to be beneficial and useful in improving patients’ independence and mobility.

## 1. Introduction

Coronavirus disease 2019 (COVID-19) is an infectious disease caused by SARS-CoV-2 that mainly affects the respiratory system [1]. While the majority (80%) of the people infected with COVID-19 present mild to moderate disease [2,3], a considerable number, generally older than 65 years and with comorbidities such as diabetes, cardiovascular disease, chronic lung disease, or obesity, can have very serious sequelae [4,5]. Of those requiring hospitalization, a high portion (20%) require management in the intensive care unit (ICU), often due to acute respiratory distress syndrome (ARDS) [6,7]. The consequences for ICU survivors can be long-lasting involving their physical, psychological, and cognitive health [8]. For the constellation of these symptoms and their long-term sequelae, the term “post-ICU syndrome” has been coined to refer to “new or worsening impairment in physical, cognitive, or mental health status arising after critical illness and persisting beyond discharge from the acute care setting” [9]. However, not only critical but also patients with severe COVID-19 can present multiorgan involvement, with cardiovascular, neurological, or skeletal muscular manifestations in addition to lung disease [10,11].

Additionally, in the post-acute and long-term phases, a wide range of nonpulmonary manifestations and complications have been reported. Patients with a severe or critical COVID-19 illness in the acute phase develop a myriad of functional deficits that impact their physical performance and ability to return home, including cognitive dysfunction, reduced aerobic capacity, fatigue, muscle weakness, anxiety, and depression [12,13,14,15,16,17]. Hence, there is a clear need for specific rehabilitation approaches for these patients to improve their health outcomes [16].

Since the outbreak of COVID-19, experts and international associations have proposed recommendations for the treatment of the affected people [18]. In line with the common guidelines for critically ill patients published by the National Institute for Health and Care Excellence (NICE) [19], many experts have recommended the prompt initiation of a multicomponent rehabilitation program based mainly on physical aerobic exercise and endurance training. The coordinated international task force composed of the European Respiratory Society and the American Thoracic Society proposed to begin a low-/moderate-intensity physical exercise in the first weeks of hospitalization in COVID wards [18]. According to a review published in 2020, patients with COVID-19 should follow a regular program of aerobic exercise in the form of cycling or walking for 20–60 min at an intensity of 55–80% maximal oxygen uptake (VO_2max_) or 60–80% of maximum heart rate (HR_max_). With the spread of COVID-19 and the identification of post-COVID-19 syndrome, other authors highlighted the importance of rehabilitation also for patients with COVID-19 sequelae [20]. Nevertheless, although many of the published studies were based on expert consensus opinions and statements suggesting that early rehabilitation is the most essential approach for patients with COVID-19, to date, there are very few randomized controlled trials (RCTs) regarding respiratory rehabilitation and telemedicine, and only one regarding aerobic exercise [21,22]. The American College of Sports Medicine (ACSM) defines aerobic exercise as any activity that uses large muscle groups, can be continuously maintained, and is rhythmic in nature [23]. A recent review has underlined that the studies conducted propose intervention programs that are not RCTs and are highly heterogeneous: The described aerobic exercises involved different modes of aerobic training, different durations ranging between 5 and 30 min, and intensities assessed in different ways or not always reported [24]. For example, while Foged et al. [25] reported the safety of performing three bouts of high-intensity interval training in individuals recently recovered from severe COVID-19, Mohamed and Alawna [26] indicated that 2 weeks of moderate-intensity aerobic training decreased the severity of symptoms and improved immune functions in young adults with mild to moderate COVID-19. More recently, Araújo et al. [27] found that a cardiopulmonary rehabilitation program consisting of continuous moderate-intensity aerobic and resistance training in patients with severe COVID-19 improved respiratory muscle strength, lung function, exercise tolerance, fatigue, and quality of life. However, no control group and no detailed training parameters were presented. To date, only Nambi et al. [28] have conducted an RCT investigating the effects of two different intensities of aerobic exercise in addition to resistance training in people with post-COVID-19 sarcopenia. Interestingly, they found that clinical and psychological measures improved more after 8 weeks of low-intensity training than after high-intensity aerobic training. However, although this study was of great interest, it was limited by its all-male cohort and the absence of a control group (i.e., with no training), which could have provided information on the absolute benefit or harm of training.

Therefore, the primary aim of this study was to conduct an RCT to study the feasibility of low- to moderate-intensity aerobic exercise training, in addition to standard rehabilitation, in patients with the sequelae of interstitial pneumonia due to COVID-19. In particular, we sought to verify if patients could tolerate this kind of physical activity and if there were any adverse effects. The secondary aim was to evaluate if the addition of aerobic exercise could be effective in improving functional capacity and gait performance compared with standard physiotherapy alone.

## 2. Methods

This prospective RCT was conducted between October 2021 and September 2022, at the Istituti Clinici Scientifici Maugeri IRCCS, Institute of Veruno, Italy. The study protocol was approved by the Ethics Committee of the Istituti Clinici Scientifici Maugeri, Italy (approval number #2592CE) and was registered in clinicaltrials.gov (ID number NCT05302973).

The patients were recruited from those admitted to the post-COVID ward of the Department of Physical Medicine and Rehabilitation with a diagnosis of interstitial pneumonia due to COVID-19 after being discharged from the COVID-19 ward following two negative nasopharyngeal swab tests for SARS-CoV-2. In particular, we enrolled patients with a negative swab but who had experienced severe or critical COVID-19 illness in the acute phase. As reported by the World Health Organization [29], people with severe COVID-19 present oxygen saturation (SpO_2_) < 90% at room air, and signs of pneumonia and severe respiratory distress; conversely, individuals with a critical illness are those with ARDS or respiratory failure requiring the provision of life-sustaining therapies, septic shock, and/or multiple organ dysfunction. The other inclusion criteria were functional independence before the COVID-19 infection and a Mini-Mental State Examination (MMSE) score > 24. The exclusion criteria were positivity to COVID-19, patients living in nursing homes or not autonomous prior to COVID-19, the presence of moderate or severe heart disease (Grade III or IV, New York Heart Association), the presence of neurological disease, low compliance, patients with low rehabilitation needs (total score at admission >100 on Functional Independence Measure (FIM)), a length of stay in acute care of >100 days, and terminal illness (life expectation <6 months). All the eligible patients received written and oral information about the study and gave their written informed consent before enrollment.

The recruited patients were randomly assigned to the experimental or control group through a randomization process (STATA command “ralloc”) conducted by an institutional statistician not involved in the enrollment, assessment, or treatment of the patients. Data were collected at the baseline and at follow-up 2 weeks later (i.e., at the end of 10 sessions). The two physical therapists who evaluated all the patients both at the baseline and at follow-up were blinded to patients’ group allocation.

### 2.1. Outcome Measures

The participants’ demographic and clinical data, including the presence of comorbidities assessed through the Severity Index and the Comorbidity Index of the Cumulative Illness Rating Scale (CIRS) [30], were collected at the time of entry in the post-COVID ward.

The primary outcome of this study was the feasibility of the intervention (the addition of aerobic training to standard rehabilitation), which included eligibility rate, recruitment rate, number of dropouts, adverse events, and adherence. The eligibility rate was defined as the number of patients eligible as a percentage of the total number of patients admitted in the study period with interstitial pneumonia due to severe or critical COVID-19 and discharged from the COVID-19 ward. The recruitment rate was defined as the number of patients recruited among those eligible. The dropout rate was the number of participants among those enrolled who did not complete the training due to nonmedical reasons. Adverse events were classified as serious adverse events (defined as death or life-threatening events requiring hospitalization), events linked to the intervention, and events leading to the interruption of the intervention. Finally, adherence, assessed only in the training group, was expressed both as attendance and compliance rates [31]. Attendance was defined as the number of completed sessions divided by the number of planned sessions. Compliance was defined as the number of sessions in which subjects reached the prescribed goal in terms of duration (% sessions performed reaching the goal of 30 min) and prescribed intensity (% sessions performed at 55–85% of HR_max_).

Secondary outcomes were as follows: the timed-up-and-go (TUG) test, the muscle torque of the knee extensors of both legs, the handgrip test, the 30 s sit-to-stand (30 s STS) test, the Cumulated Ambulation Score—Italian version (CAS-I), and the FIM.

#### 2.1.1. Timed-Up-and-Go (TUG) Test

This is a physical functional measure in which subjects stand up from a chair, walk 3 m to a horizontal line marked with tape on the floor, turn around, walk back, and sit down at their comfortable pace [32]. The participants could use their usual walking aid but assistance was not permitted. After a practice trial, two timed trials were performed, and the time necessary to complete them was recorded with a stopwatch and then averaged.

#### 2.1.2. Muscle Torque of Knee Extensors

This assessment was performed with the use of a handheld dynamometer (Lafayette Manual Muscle Tester, model 01163, Lafayette Instruments, Lafayette, IN, USA). The patients were required to perform a sub-maximal contraction in knee extension, followed by two maximal contractions during which the physiotherapist gave verbal encouragement [33]. Standardized instructions were used: “When I instruct you to start, push against the dynamometer as hard as you can until I tell you to stop”. The evaluation was performed first on one limb, then on the other, with a 30 s pause between one contraction and the next. The mean value of the two right-leg and left-leg tests was recorded. The measurements were converted from kg to (N·m)/kg using the formula: [(peak force (kg) × 9.81) × distance from joint (m)]/weight (kg) [33].

#### 2.1.3. Handgrip Strength

The handgrip strength was measured bilaterally with a calibrated hand dynamometer (Jamar^®^ hydraulic hand dynamometer, Sammons Preston Incorporated, Bolingbrook, IL, USA). In accordance with the recommendations of the American Society of Hand Therapists, the subjects were seated with feet flat on the floor, the tested arm adducted against the body in neutral rotation, the elbow in 90° of flexion, and the forearm in neutral rotation pronation/supination [34]. Two trials for each arm were performed; the average of the right and left force was recorded. The handgrip strength is a valid and reliable measure of the total force from the upper limb muscles. It is demonstrated to be highly predictive of frailty, future functional limitations, and disability [35].

#### 2.1.4. The 30 s Sit-to-Stand Test (30 s STS)

The 30 s STS is a widely used measure of lower body muscular strength [36] that captures the number of stands a person can complete in 30 s without using the arms. It was administered using a folding chair without arms, with a seat height of about 43–46 cm. When performing the test, the chair was placed against a wall to prevent it from moving. The patients were asked to perform the repetitions of standing upright and then sitting down in the same position at a self-paced speed (safe and comfortable) as many times as possible for 30 s, without using their arms and fully sitting between each stand. The number of completed stands and the SpO_2_ before and after the test were recorded.

#### 2.1.5. Cumulated Ambulation Score—Italian Version (CAS-I)

The CAS-I is a 3-item scale assessing three mobility skills: (1) getting in and out of bed, (2) sitting to standing from a chair with armrests, and (3) walking indoors with the use of appropriate walking aids [37]. Each activity was scored on a 3-point ordinal scale from 0 to 2 (0: not able to, despite human assistance and verbal cueing; 1: able to do so with human assistance and/or verbal cueing from one or more persons; 2: able to do so safely without human assistance or verbal cueing, but the use of a walking aid allowed). The total score range was 0 to 6.

#### 2.1.6. Functional Independence Measure (FIM)

This scale is widely used to rate patients’ ability in performing activities of daily living (ADLs) [38]. It is composed of 18 items (13 motor and 5 cognitive), each of which is scored from 0 (total dependence) to 7 (total independence). The total FIM score ranges from 18 (reflecting complete functional dependency) to 126 (complete functional independence).

### 2.2. Rehabilitation Protocol

All the patients (experimental and control group) underwent a standard inpatient rehabilitation program of 10 sessions (50 min/day, 5 days/week) administered by 4 regular ward physiotherapists skilled in both orthopedic and respiratory rehabilitation. The main components of the program were as follows: mobilization, upper and lower limb strengthening, balance exercise, walking training, and respiratory muscle training [39].

In addition to this, only those patients in the experimental group also underwent aerobic training in line with the criteria of the Official American Thoracic Society/European Respiratory Society Statement [40] and the recommendations of the ACSM [41,42]. Aerobic training was performed with an arm crank ergometer (MOTOmed^®^ Reck, Betzenweiler) for 30 min/day, 5 days/week, for a total of 10 sessions. Each training session consisted of 5 min warm-up, 20 min of training, and 5 min cool-down. The patients were encouraged by the physiotherapist to cycle at a fixed frequency of 60 rpm, helped by the feedback from the ergometer display. The intensity of the training was adjusted so as to maintain the rating of perceived exertion (RPE) between 11 and 14 of the Borg Scale, or within 55–85% of HR_max_. HR_max_ was calculated with the following formula: 220-age [43]. The intensity was progressively increased within and across sessions by increasing the workload according to the patient’s abilities. We also examined the energy expenditure estimating the metabolic equivalents (METs). The estimated METs were calculated from the power of the arm crank ergometer exercise, expressed in Watts, combined with the patient’s anthropometric data, according to the ACSM equations [41].

Before each training session, vital signs such as blood pressure, SpO_2,_ and HR were evaluated, and oxygen therapy was recorded. If the blood pressure was >160/100 mmHg and HR >100 or <50 beats per minute, the participant was not allowed to undergo aerobic exercise on that particular day [44]. During each session, training intensity was monitored via HR and RPE, while SpO_2_ was continuously measured with a pulse oximeter.

Supplemental oxygen therapy was prescribed by the ward specialist (a pneumologist or a physiatrist) for both rest and physical activity. Physiotherapists were allowed to titrate oxygen supplementation within the limits imposed by the physician’s prescription. To assist physiotherapists in oxygen titration, the algorithm proposed by the Cardiovascular and Pulmonary Section of the American Physical Therapy Association was used [45]. During aerobic training, the target SpO_2_ was set at 90% or above in order to avoid severe exercise-induced hypoxemia [46].

### 2.3. Sample Size

The sample size was estimated based on the TUG test, a key clinical secondary outcome, as the effectiveness in improving mobility is considered important to power trials in rehabilitation settings [47]. We expected a mean difference between the control and training groups of at least 3.01 s, which corresponds to the minimal detectable change in the TUG test in subjects with respiratory disease [48]. We imposed a standard deviation of 2.5 s based on preliminary data of patients with COVID-19 [17]. Setting the significance at 5% and power at 80%, a sample size of 13 patients per group was required [49]. Estimating a dropout rate of 20%, we finally established a sample of 30 subjects [50]. The analysis followed the intention-to-treat principle. The missing data from dropouts were replaced with the mean of the missing subjects’ own group. 

### 2.4. Statistical Analysis

The baseline differences between the groups were examined using the chi-square (χ^2^) test for categorical data and the Mann–Whitney U test for the interval and continuous variables not normally distributed. The Wilcoxon signed-rank test was run to compare the pre–post rehabilitation scores within each group. The continuous and interval variables assessed after rehabilitation were compared using χ^2^ for the categorical variable test and the analysis of covariance (ANCOVA), with the baseline score as a covariate, for ordinal variables. When ANCOVA gave significant results, the Tukey post hoc test was performed. Finally, the clinical meaning of differences between the two intervention groups was assessed through the calculation of partial eta-squared (η*p*^2^) as a measure of effect size. The values of η*p*^2^ of 0.01, 0.06, and 0.14 were considered the small, medium, and large effect sizes, respectively [49].

To evaluate the effect of aerobic exercise on the training variables (HR_max_, RPE, Watts, and MET), a one-way repeated-measure ANOVA with one independent factor (daily training sessions) was run. When ANOVA gave a significant result, the post hoc Tukey test was conducted to assess the differences between the sessions. Statistical significance was set at *p* < 0.05. Statistical analysis was performed using Statistica (StatSoft Inc., Tulsa, OK, USA).

## 3. Results

### 3.1. Feasibility

**Figure 1:**
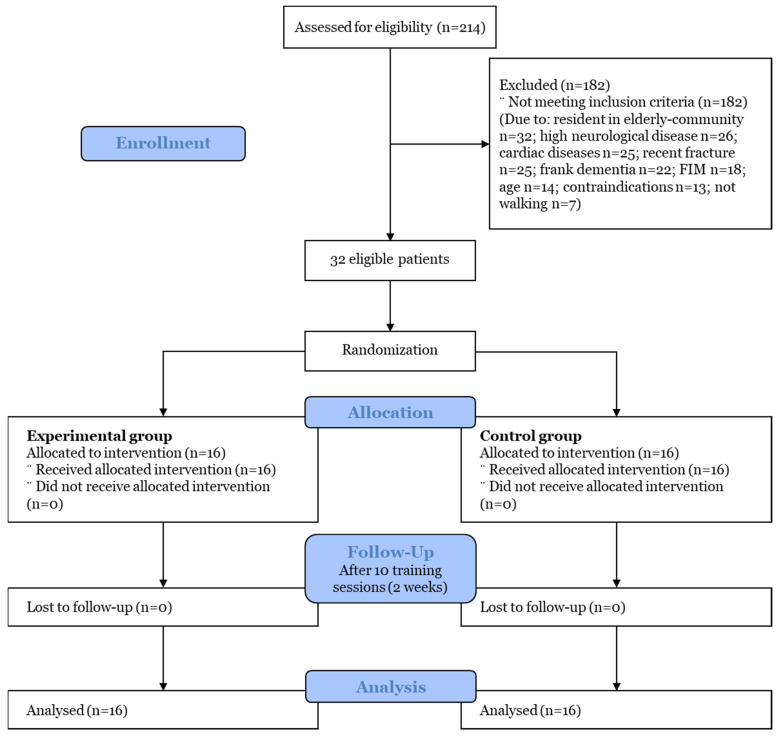
CONSORT flow diagram.

The participant flow and reasons for dropout are presented in Figure 1. Among the 214 patients admitted to the post-COVID ward with a diagnosis of interstitial pneumonia, 32 patients with subacute COVID-19 were considered eligible, and all of them agreed to participate in this study (eligibility rate: 32/214, 15%; recruitment rate: 32/32, 100%). The patients were subsequently randomized and assigned to the training or control group. The baseline characteristics of the 32 participants, divided based on treatment group, are summarized in Table 1. No significant difference was found between the groups in terms of demographic, clinical, or performance data. Moreover, among the 32 total patients, 26 patients were not fully vaccinated against COVID-19. However, the clinical characteristics were not different among the fully vaccinated vs. unvaccinated patients, maybe due to the small sample size. According to CIRS categories, the most frequent somatic symptoms were found to be hypertension (31%), endocrine–metabolic disease (19%), and vascular disease (11%).

During the study period, no participant dropped out, and no severe adverse events were reported. However, three participants were not able to complete the 30 min aerobic training in the first and second sessions due to high levels of fatigue (RPE > 14), while one patient could not achieve the goal of 30 min of aerobic exercise in four sessions due to arm muscular pain and dyspnea. Regarding adherence to aerobic training, the mean attendance rate was 95.6 ± 12.3%. Specifically, 2 participants attended only 6 and 7 of the 10 sessions, respectively, while the remaining 14 attended all 10 sessions. The reason why the two patients did not attend all the sessions was that their blood pressure and HR did not allow them to undergo aerobic training on those days. Due to the above adverse events, the mean compliance rate with the duration of aerobic training was 93.8 ± 12.0%. Finally, the mean compliance rate with the intensity of aerobic training was 87.9 ± 22.6%. In particular, two participants achieved the set goal of the intensity of 55% of HR_max_ in less than half of the total sessions, three patients achieved it within seven sessions, while the other eleven reached the intensity goal by the end of the attended sessions.

**Figure 2:**
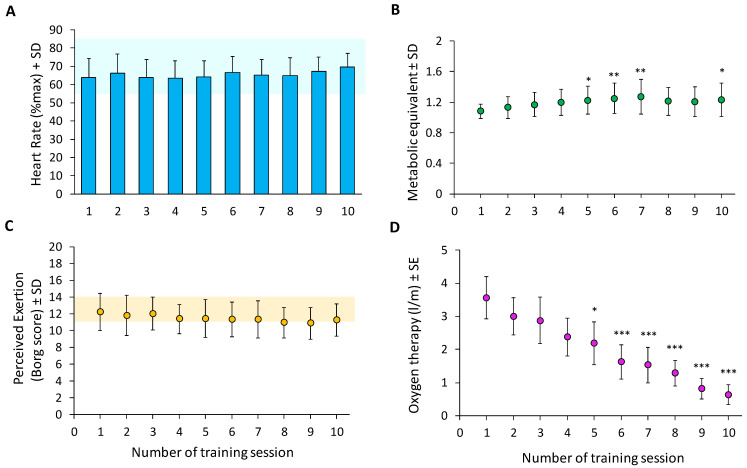
Characteristics of aerobic exercise in training group (n = 16).Both the heart rate (**A**) and the rating of perceived exertion assessed with the Borg Scale (**C**) did not significantly change across the training sessions; this demonstrated that patients with subacute COVID-19 performed a moderate-intensity level of activity, as indicated by the light blue (**A**) and orange bars (**C**), respectively. The metabolic equivalents (METs) (**B**) partially improved across the training sessions, but their mean value across the sessions remained always below the level of 3 METs, thus suggesting that the participants performed a light-intensity physical activity. Oxygen therapy (**D**) significantly decreased from session 5 to 10, compared with day 1 of the training.Asterisks refer to the comparison between subsequent days and the first day of the training. *, *p* < 0.05; **, *p* < 0.005; ***, *p* < 0.0005.

Most of the training details are reported in Figure 2. The %HR_max_ (Figure 2A) and the RPE (Figure 2C) did not significantly change across the training sessions, indicating that the participants in the experimental group were performing low- to moderate-intensity aerobic exercise. In fact, the mean %HR_max_ was maintained in the range of 55–85%, while the RPE was reported to be between 11 and 14. Nevertheless, the average MET indicated that the patients mainly performed light-intensity physical activity, as the values of all sessions were below the threshold of 3 METs (Figure 2B); however, when compared with the first session, an increase in the mean activity level according to METs was observed in sessions 5, 6, 7, and 10.

Finally, a significant reduction in oxygen therapy (Figure 2D) was observed across the training sessions. While at session 1, the patients required a mean of 3.6 L/m supplemental oxygen, by the last session, they required only 0.6 L/m. In particular, when compared with day 1 of the training, the decrease observed on day 5 was significant (*p* < 0.05) and even more so on day 10 (*p* < 0.0005). Despite reducing the level of oxygen therapy, the level of SpO_2_ always remained in the range recommended by the guidelines. On day 1, the mean SpO_2_ at rest was 96% (± 3%), while on day 10, after the aerobic training sessions, it was 95 ± 1%; the mean SpO_2_ before and after aerobic training was 94 ± 2% and 93 ± 3%, respectively.

### 3.2. Secondary Outcomes

Pre- and post-intervention values are shown in Table 2.

After rehabilitation, the mean muscle torque of the knee extensors significantly improved in both groups (Wilcoxon test, *p* < 0.0005 and *p* < 0.005 for the experimental and control groups, respectively) with no significant difference between them (ANCOVA, *p* = 0.07). Post-training, the mean handgrip strength increased only in the experimental group (Wilcoxon test, *p* < 0.005), while no difference was found in the control group (Wilcoxon test, *p* = 0.26); no significant difference was found between the groups (ANCOVA, *p* = 0.09).

The total FIM score significantly improved in both groups (Wilcoxon test, *p* < 0.0005), but the difference was significantly greater in the experimental group (15 points for control vs. 24 points for the experimental group, ANCOVA, *p* < 0.05; post hoc, *p* < 0.05).

The CAS-I total score increased in both groups (Wilcoxon test, *p* < 0.005) after the 10 days of rehabilitation, with a mean improvement of 1.1 for the experimental group and 1.4 for the control group, showing a significant difference between the groups (ANCOVA, *p* < 0.005; post hoc, *p* < 0.005).

Other secondary outcome measures improved after training in both groups, though without showing significant differences between the groups. At the baseline, only 12 out of the 16 patients in the experimental group and 11/16 in the control group were able to perform the TUG test (χ^2^ = 0.69); post-training, 15 patients of the experimental group completed the TUG test with a mean time of 13.7 ± 4.9 s, while all the participants in the control group were able to perform it with a mean time of 17.5 ± 8.4 s (ANCOVA, *p* = 0.49). The 30 s STS significantly improved in both groups (Wilcoxon test, *p* < 0.005 and *p* < 0.05 for the experimental and control groups, respectively) with no significant difference between them (ANCOVA, *p* = 0.33).

## 4. Discussion

This randomized controlled trial is the first to investigate the feasibility, safety, and effectiveness of the addition of aerobic training to standard rehabilitation in survivors of COVID-19. The results suggest that low- to moderate-intensity aerobic exercise training is feasible in subacute people with a diagnosis of severe or critical COVID-19 illness in the acute phase. In our study, only 15% of the patients admitted to the post-COVID ward were eligible for recruitment in the trial. This low eligibility rate was mostly related to two factors: the restrictive inclusion criteria and the heterogeneity of the sample of patients commonly admitted to a post-COVID ward [51]. In fact, most of those excluded had cognitive impairment or other important cardiovascular or neurological diseases that could have interfered with the training, or they were nursing home residents before hospitalization [52]; conversely, others had low rehabilitative needs and were discharged within a few days after admission. Nevertheless, all those who were eligible accepted to participate in the trial, with an attendance rate of 100%, and no dropouts were recorded, suggesting that the inclusion criteria were adequate. Such a high attendance rate could be because, in addition to respiratory symptoms, patients with severe or critical COVID-19 also experienced limb muscle weakness, muscle atrophy, and impairment in performing ADLs [53,54,55,56]. A systematic review of outcomes from previous coronavirus epidemics highlighted that 41% of patients had a reduced aerobic capacity at 3 months post-illness [54]. Therefore, patients feel the importance of rehabilitation for regaining their physical performance.

The target of aerobic training in the present study was defined as 30 min of exercise at an intensity of 55–85% HR_max_, in keeping with the recommendations of ACSM for low- to moderate-intensity aerobic exercise in deconditioned individuals and adults [41]. To our knowledge, this is the first study to demonstrate that patients with severe or critical COVID-19 actually managed to achieve and maintain the target duration and intensity of aerobic exercise during the sessions. The adherence to the 10 sessions of low- to moderate-intensity aerobic training was high, with the participants attending about 96% of the programmed sessions, and only a few mild adverse events were observed. Nevertheless, due to the nature of the COVID-19 condition, i.e., severe or critical illness in the subacute phase, not all participants were able to complete all the sessions or achieve the prescribed intensity. It has been well documented that people after COVID-19 pneumonitis are characterized by fatigue and physical deconditioning, which is also the most frequent cause of their impaired level of VO_2max_ [2,57,58]. In particular, in those who experienced ARDS and had a prolonged ICU stay, exercise capacity, respiratory function, and neurological impairments may have been affected, leading to a post-intensive-care syndrome characterized also by muscle weakness with consequences on the quality of life [54,55,56]. Not surprisingly, the two patients who did not complete the full 30 min of aerobic training on the first 4 days (n = 1) or did not reach the prescribed intensity in most of the sessions (n = 1) were those with the longest length of stay in the ICU.

The addition of aerobic exercise performed with an arm crank ergometer appears to be beneficial for people with subacute COVID-19. Previous studies have reported that arm aerobic exercise can be an effective training modality among healthy older people and patients with hip fractures, peripheral arterial disease, and post-stroke [33,59,60,61]. The benefits gained regard not only the cardiovascular function but also the physical functional performance, such as walking, balance, mobility, and motor acquisition since several lines of evidence suggested that a single bout of moderate-intensity aerobic exercise may also be enough [62]. In our study, people with post-acute COVID-19 with marked motor weakness, even if they were mostly free of pre-morbid neurological disability, showed improvement in several measures after aerobic exercise training; in particular, their functional outcome measures—FIM and CAS-I—significantly increased more than that the measures observed in those who performed only conventional rehabilitation. We chose to use FIM because it examines both the physical function and the activities of daily life, as a result of the severity of the acute disease (mobility restrictions due to dyspnea, the presence of delirium, etc.) but also the chronic conditions exacerbated or not exacerbated by acute disease [63]. In a previous study investigating the effectiveness of pulmonary rehabilitation in patients with severe and critically ill COVID-19, there was a gain of about 18 points in the FIM total score by the end of the 22 days of post-acute inpatient rehabilitation [64]. The improvement we found in the control group seems to be in line with this gain, with the patients having increased their score by about 15 points in only 14 days. On the other side, the patients in the experimental group obtained a still greater improvement—about 24 points—which emphasizes the important contribution of the 30 min sessions of aerobic training for reaching a higher level of functional independence.

Conversely, the mean time necessary for performing the TUG test and the 30 s STS improved in both the control and experimental groups, without any differences between them. However, more repetitions of the 30 s STS in both groups at follow-up were still below the normative value for 70 years old elderly [65]. These results are in line with our previous study [33]. Notably, in patients with recent femur fractures, aerobic training with an arm crank ergometer did not result in a greater improvement in performance compared with those who underwent only standard rehabilitation and did not reach the normal range in the brief rehabilitative period. However, the 30 s STS is strongly correlated with lower limb strength [66]. In fact, this test has been proposed as a measure for assessing functional performance and lower limb strength in adults, in particular those discharged from the hospital after a severe illness in the ICU and COVID-19 survivors in the early stages of rehabilitation [66,67,68]. It is, therefore, not surprising that, in addition to the improved performance on the 30 s STS in both groups, at the end of the study, we also found improvement in the knee extensor strength in both groups. Due to the absence of difference between the groups, it is reasonable to suppose that the increase in strength was due to the exercises performed during standard rehabilitation rather than aerobic training. Moreover, it is known that ICU-acquired weakness in patients with acute lung injury has been found to persist in 14% of patients at 12 months [54]; it is logical to suppose that the 30 s STS and lower limb strength might also be improved in the subsequent discharge period. Thus, incomplete recovery at discharge may imply the need to continue with outpatient rehabilitation.

In the handgrip test, while both groups improved at the end of the training, the improvement was significant only in the experimental group. Nambi et al. [28], in their study comparing low- and high-intensity aerobic training, found no difference in the handgrip test between the groups, but their findings suggest that, with a longer period of training, a difference could have emerged. In fact, the difference in the handgrip test between the two groups became significant after 8 weeks of training, even if the low-intensity training group showed a trend of greater improvement in the first weeks of training. Moreover, in our study, aerobic exercise was performed with an arm crank ergometer. Thus, it is reasonable to suppose that the participants of the experimental group would show more improvement in a test using the upper limbs. In fact, in a sample of patients with stroke trained for 8 weeks with an arm crank ergometer, a bilateral increase in grip strength was found, accompanied by increased muscle activation in the wrist flexors, such as the flexor carpi radialis [61]. All in all, the present study is in line with other studies that found that the major functional consequences after the COVID-19 acute period seem to be motor [69]. Future studies could try to determine whether this motor deficit is attributable to only deconditioning, given by prolonged hospitalization, or to direct aggression in the neurological pathways of COVID-19.

### Limitations

Our study has some limitations. First, the intensity of the aerobic exercise was based on the %HR_max_ and RPE assessed with the Borg Scale rather than calculated from cardiopulmonary exercise testing. However, most of the patients were so deconditioned that they exercised at 0–1 Watts during training.

Second, we cannot state with certainty if the higher improvement observed in the experimental group was due to the aerobic training per se or, more simply, to the additional 30 min/day of rehabilitation. Third, due to the usual length of stay of patients in the post-COVID ward, we considered only 10 training sessions in the protocol, which may not have been sufficient to achieve the full expected effects of the exercise program. Finally, although the participants’ history of vaccination against COVID-19 was recorded, the limited sample of enrolled patients who were fully vaccinated did not allow us to perform any subgroup analysis. We do not exclude that the response to rehabilitation in those vaccinated and unvaccinated was different; future studies may be conducted to analyze these two populations separately.

## 5. Conclusions

In conclusion, the results of this study indicate that the addition of arm-cranking aerobic exercises to standard rehabilitation is feasible and safe for patients with subacute COVID-19 illness, without adverse events. Moreover, it may be effective in improving the functional outcome measures, in particular independence and mobility, during inpatient rehabilitation.

## Figures and Tables

**Table 1:** Baseline characteristics of participants (n = 32).

Characteristics	Experimental Group (n = 16)	Control Group (n = 16)	*p*-Value
Demographic			
Age (years)	70.6 ± 12.2	70.5 ± 9.2	0.88
Sex	6 F; 10 M	7 F; 9 M	0.72
Clinical			
Current occupation	5 E; 11 U	3 E; 13 U	0.41
Current smoker	5 Y; 11 N	4 Y; 12 N	0.69
Fully vaccinated against COVID	2 Y; 14 N	4Y; 12N	0.37
CIRS (Severity Index)	1.8 ± 0.3	1.9 ± 0.4	0.58
CIRS (Comorbidity Index)	4.1 ± 1.7	4.5 ± 1.7	0.54
Body mass index (kg/m^2^)	25.7 ± 3.4	26.9 ± 4.9	0.40
Resting heart rate (beats/min)	84.5 ± 14.5	85.6 ± 15.0	0.73
Oxygen therapy (L/m)	3.8 ± 2.6	2.6 ± 3.6	0.07
Oxygen saturation (%)	95.3 ± 2.2	94.1 ± 2.8	0.30
Time in ICU (days)	7.9 ± 15.5	5.8 ± 10.9	0.67
Diagnosis in acute infection	4 C; 12 S	4 C; 12 S	1.0
Time between 1st positive swab and randomization (days)	27.9 ± 7.4	33.8 ± 11.9	0.31
Length of stay in post-COVID ward (days)	13.8 ± 2.6	13.8 ± 1.8	0.98
Performance			
FIM (total score)	80.3 ± 12.7	77.0 ± 14.7	0.39
CAS-I (total score)	4.3 ± 1.4	3.7 ± 1.7	0.37
30 s STS (n)	4.1 ± 3.6	2.9 ± 4.3	0.27
TUG test (s)	21.8 ± 9.3	21.3 ± 6.5	0.81
Handgrip (kg)	20.8 ± 5.1	20.4 ± 7.4	0.79
Knee extension strength (Nm/kg)	86.8 ± 25.7	76.4 ± 28.3	0.20

Abbreviations: C, critical; CAS-I, Cumulated Ambulation Score—Italian version; CIRS, Cumulative Illness Rating Scale; E, employed; F, female; FIM, Functional Independence Measure; ICU, intensive care unit; M, male; MMSE, Mini-Mental State Examination; N, no; S, severe; STS, sit-to-stand; TUG, timed up and go; U, unemployed; Y, yes.

**Table 2:** Secondary outcomes (n = 32).

	Training Group (n = 16)			Control Group (n = 16)			Comparison	
	Baseline	Follow-Up	*p*-Value	Baseline	Follow-Up	*p*-Value	*p*-Value	η*p*^2^
	Mean ± SD	Mean ± SD		Mean ± SD	Mean ± SD			
TUG test (s)	21.8 ± 9.3	13.49 ± 4.9	**<0.005**	21.3 ± 6.5	17.5 ± 8.4	**<0.005**	0.49	0.02
Muscle torque of knee extension (Nm/kg)	86.8 ± 25.7	110.6 ± 28.3	**<0.0005**	76.4 ± 28.3	91.3 ± 29.7	**<0.005**	0.07	0.11
Handgrip strength (kg)	20.8 ± 5.1	23.3 ± 5.3	**<0.005**	21.2 ± 7.4	21.9 ± 6.9	0.26	0.09	0.10
30 s STS (n)	4.1 ± 3.6	7.3 ± 3.3	**<0.005**	2.9 ± 4.3	5.8 ± 3.6	**<0.05**	0.33	0.03
CAS-I (total score)	4.3 ± 1.4	5.4 ± 0.3	**<0.005**	3.8 ± 1.5	5.2 ± 0.9	**<0.005**	**<0.005**	0.22
FIM (total score)	80.3 ± 12.7	104.7 ±12.7	**<0.0005**	77.0 ± 14.7	92.3 ± 11.7	**<0.0005**	**<0.05**	0.15

Abbreviations: 30 s STS, 30 s sit-to-stand; CAS-I, Cumulated Ambulation Score—Italian version; CIRS, Cumulative Illness Rating Scale; FIM, Functional Independent Measure; SD, standard deviation; TUG, timed up and go; η*p*^2^, partial eta-squared; bold highligted the statistical significant result.
